# Metal and metalloid biorecovery using fungi

**DOI:** 10.1111/1751-7915.12767

**Published:** 2017-07-11

**Authors:** Xinjin Liang, Geoffrey Michael Gadd

**Affiliations:** ^1^ Geomicrobiology Group School of Life Sciences University of Dundee Dundee DD1 5EH UK

## Abstract

Bioleaching is a proven bioprocess for metal recovery by solution from solid matrices, while a bioprecipitation or biomineralization approach is of potential for biorecovery from solution. Fungi can directly and indirectly mediate the formation of many kinds of minerals, including oxides, phosphates, carbonates and oxalates, as well as elemental forms of metals and metalloids such as Ag, Se and Te. Fungal capabilities may offer a potentially useful contribution to biotechnological and physico‐chemical methods for metal recovery.

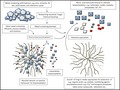

## Sustainability of metal supply

The application of microbial systems for metal and metalloid bioprocessing and biorecovery has received increasing attention in recent years, with renewable energy supplies and sustainable environmental concepts becoming new trends in many industries. Demands from environmental technologies and applications for clean and efficient energy production and usage rely on a range of raw materials, of which metals are of fundamental and strategic importance (Vaughan *et al*., 2002; Graedel *et al*., 2015; Table 1). However, many important metal resources are now threatened by overexploitation, inadequate recycling and reclamation, and geopolitical issues. Industrial consumption of metals and minerals has increased significantly in recent decades and rising population growth ensures that demand will accelerate. The growth of highly populated mega‐cities has also exacerbated problems of metal recycling and reclamation and has given rise to the concept of ‘urban mining’ – the recovery of important elements from urban waste (Cossu *et al*., 2012; Lyons and Harmon, 2012). In addition, the increasing need for energy production from renewable resources, such as solar and wind power, and energy‐efficient electronic materials, including those used in computers, mobile phones and televisions, are highly dependent on a wide range of metal and mineral resources. Such ‘E‐tech elements’ include cobalt, platinum group metals (PGM) and rare earth elements (REE), as well as metalloids like selenium and tellurium (Table 1). Some of these elements are already in short supply, can be difficult to recover by conventional mining and extraction processes and may be found in significant amounts in only a small number of geographic locations rendering the supply chain vulnerable to economic and political forces (Natural Environment Research Council 2013). The EU is almost wholly dependent on imported supplies of such elements. It is therefore accepted that there is an urgent need to improve the global security of supply of important elements at the same time balancing new mining processes with minimizing environmental impact such as pollution and increased greenhouse gas emissions. In the many worldwide initiatives designed to address this problem, microbial bioprocessing is seen as an essential component of the approaches that may be used to improve metal biorecovery (Natural Environment Research Council 2013; Pollmann *et al*., 2016). Compared with conventional chemical and physical recovery, biorecovery of E‐tech elements using microbial systems may have advantages in low energy requirements, low carbon emissions and cost, providing impetus towards development of novel biotechnologies for sustainable E‐tech metal(loid) biorecovery.

**Table 1:** Important and critical metals and metalloids for new and developing technologies that have been identified as having significant future risks in security of supply, extraction, recycling and geopolitical threats. Most are the subject of growing research on the contribution of microbial metal and mineral transformations for their bioprocessing and biorecovery

Metal/Element groups	Elements	Reference
Platinum Group Metals (PGM)	Iridium, Osmium, Palladium, Platinum, Rhodium, Ruthenium	–
Rare Earth Elements (REE)	Cerium, Dysprosium, Erbium, Europium, Gadolinium, Holmium, Lanthanum, Lutetium, Neodymium, Praseodymium, Promethium, Samarium, Scandium, Terbium, Thulium, Ytterbium, Yttrium	–
E‐tech elements	Antimony, Arsenic, Barium, Beryllium, Bismuth, Boron, Cadmium, Gallium, Germanium, Hafnium, Indium, Lithium, Magnesium, Mercury, PGM, REE, Scandium, Selenium, Silicon, Strontium, Tantalum, Tellurium, Thallium, Titanium, Tungsten, Zirconium	Natural Environment Research Council (2013)
Elements important to environmental technologies	Cobalt, Gallium, Indium, Lithium, Neodymium, Niobium, PGM, REE, Tellurium, Vanadium	Natural Environment Research Council (2013)
Borderline critical elements of potential future high risk	Lithium, Selenium, Tellurium, Vanadium	Natural Environment Research Council (2013)
Critical elements for low carbon energy technologies (in order of decreasing demand)	Tellurium, Indium, Tin, Hafnium, Silver, Dysprosium, Gallium, Neodymium, Cadmium, Nickel, Molybdenum, Vanadium, Niobium, Selenium	European Union (2011)
High risk elements for low carbon energy technologies	Dysprosium, Gallium, Indium, Neodymium, Tellurium,	European Union (2011)
Critical elements for the EU (in order of decreasing forecast demand to 2020)	Niobium, Gallium, REE, Cobalt, Indium, Magnesium, Tungsten, Chromium, Germanium, PGM, Silicon Metal, Antimony, Beryllium	European Commission (2014)
Criticality assessment of high‐importance metals to the US economy	Copper, Gallium, Indium, Lithium, Manganese, Niobium, PGM, REE, Tantalum, Titanium, Vanadium	National Academy of Sciences (2008)
Most critical metals to the US economy	Indium, Manganese, Niobium, PGM, REE	National Academy of Sciences (2008)
High risk elements vulnerable to supply and other restrictions	Arsenic, Indium, Antimony, Chromium, Manganese, Magnesium, REE, Rhodium, Selenium, Silver, Thallium	Graedel *et al*. (2015)

## Metal–mineral–microbe interactions for metal biorecovery

Metals, differentiated from non‐metals and metalloids by their physical and chemical properties, comprise > 75% of the known elements and are ubiquitous in the environment. Apart from the major industrial metals, such as copper, zinc, iron, aluminium and nickel, many other elements are of special interest because of their increasing applications in new technology (Table 1). Metalloids are a group of elements that have properties intermediate between those of the metals and non‐metals, and include commercially significant selenium, tellurium and germanium (Table 1). These elements usually have semiconductor properties and can form amphoteric oxides (Lombi and Holm, 2010).

The ability of microorganisms to change the chemical speciation of metals is well known and a significant component of natural biogeochemical cycles for metals and associated elements in rocks, minerals, soil and organic matter. A variety of mechanisms are involved in microbial attack of rocks and minerals such as physical penetration and the production of acidic and/or metal complexing metabolites such as organic and inorganic acids, and siderophores. Solubilization mechanisms provide a means of metal biorecovery from solid matrices. Bacterial bioleaching using, e.g. *Acidithiobacillus* spp., is a well‐established industrial process for several metals, e.g. Cu, from mineral ore resources (Johnson, 2014). Chemoorganotrophic bioleaching by fungi through metabolite excretion can also be effective on metal‐rich substrates. Several rare earth elements, including cerium (Ce), lanthanum (La), neodymium (Nd) and praseodymium (Pr), were released by *Aspergillus* and *Paecilomyces* spp. from monazite sand (Brisson *et al*., 2015). Chemoorganotrophic bioleaching is also applicable to biorecovery of elements from other industrial and electronic wastes (Burgstaller and Schinner, 1993). Bioreduction can also result in increased solubility, e.g. Mn(IV) to Mn(II). Metal immobilization processes may be desirable for removing metals from aqueous solution. Metals and metalloids can be immobilized as elemental or biomineral forms, e.g. Ag^0^, Se^0^, Te^0^, and manganese oxides, through redox transformations as well as by the production of metabolites such as sulfide, oxalate and CO_2_ that lead to metal precipitation as sulfides, oxalates and carbonates respectively. Biomineralization can also be mediated through release of anionic substances that combine with metals, e.g. phosphates, and carbonates, as a consequence of mineral dissolution or biodegradation of organic substrates. The variety of microbial biomineralization and bioprecipitation mechanisms provides a means of recovering elements from leachates, process streams and effluents with an additional benefit in that growth and biomass may be decoupled from the reactive liquid matrix, and recovered metal(loid)s may be in useful biomineral or elemental forms, including nanoparticles (Lloyd *et al*., 2008; Gadd, 2010). Other mechanisms of microbial metal immobilization that have received considerable attention, mostly in the context of bioremediation, are biosorption and other accumulative mechanisms but these have never successfully reached the marketplace due to several reasons including poor selectivity and the inability to compete with commercially available ion exchange resins (Gadd, 2009). Therefore, it seems bioleaching is a proven bioprocess for metal recovery by solution from solid matrices, while a bioprecipitation or biomineralization approach is of potential for biorecovery from solution.

## Fungal processes for metal mineral biorecovery

**Figure 1:**
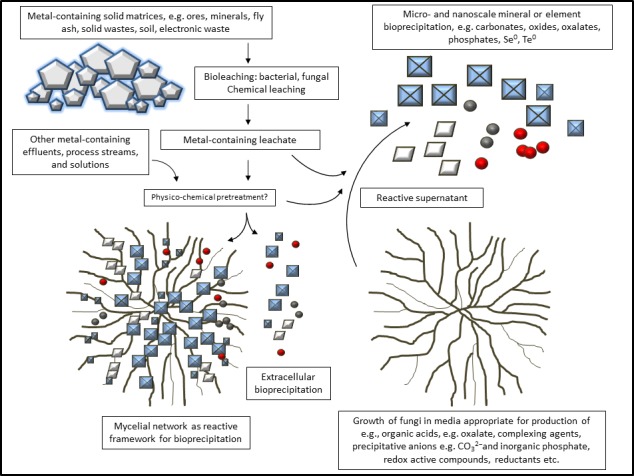
Simplified outline of fungal metal–mineral transformation processes for metal, metalloid and mineral biorecovery. Metal‐containing biological or chemical leachates or other solutions are contacted with geoactive fungal strains, with or without appropriate physicochemical treatments, e.g. pH adjustment. Mineral precipitation may occur on the mycelial network and in the external medium. Growth‐decoupled mixture of reactive fungal supernatants can lead to mineral formation in the absence of biomass. The symbols represent minerals or elemental forms and can include oxalates, oxides, carbonates, phosphates, Se^0^ and Te^0^.

Species from all microbial groups can effect geochemical transformations of metals and minerals (Gadd, 2010). This article concentrates on the fungi, a group of organisms less appreciated as geoactive agents, but nevertheless capable of many important metal and mineral transformations. Fungi interact with metals and minerals in both natural and synthetic environments, changing their chemical and physical properties, such as metal speciation and mobility, and effecting mineral dissolution and formation through a variety of metal mobilization or immobilization mechanisms (Gadd, 2007, 2010). The vast majority of fungi exhibit a branching filamentous explorative lifestyle. They are chemoorganotrophic and excrete a variety of extracellular enzymes and metabolites that interact with organic and inorganic substrates. Their geoactive properties are underpinned by their metabolism and lifestyles. The ability of fungi to solubilize insoluble metal compounds and minerals can depend on the excretion of organic acids, such as oxalic and citric acids, which not only lower the pH but also complex the metals present increasing their solubilities (Gadd, 1999; Fomina *et al*., 2005a). Fungi can also directly and indirectly mediate the formation of many kinds of minerals, including oxides, phosphates, carbonates and oxalates, as well as elemental forms of metals and metalloids such as Ag, Se and Te. Such bioprecipitation largely depends on the organism modifying its local microenvironment to create appropriate physicochemical conditions for precipitation to take place. Compared with the simpler bacterial cell form, the filamentous fungal growth habit may additionally provide more framework support and stability as a reactive network for biomineralization (Fomina *et al*., 2008; Rhee *et al*., 2012; Li *et al*., 2014; Li, Q. *et al*. 2016; Liang *et al*., 2015; Fig. 1). In addition, the production of reactive culture supernatants enables a metal biorecovery/bioprecipitation system without the complication of biomass separation (Li *et al*., 2014; Fig. 1).

## Biomineralization and bioprecipitation

Metal biorecovery from solution can be achieved through precipitation or crystallization of insoluble organic or inorganic compounds (Gadd, 2010; Gadd *et al*., 2012). Fungi are very important biodegraders of organic materials, and this can indirectly result in mineral formation where biodegradation products react with available metal species. For example, the action of phosphatase enzymes on P‐containing organic substrates results in the release of inorganic phosphate which can then precipitate with available metals, as first demonstrated in bacteria (Macaskie *et al*., 1992, 2000). Such phosphatase‐mediated metal bioprecipitation can also occur in fungi. Several fungi, including yeasts, can extensively precipitate lead or uranium phosphates on cell surfaces during growth on a source of organic phosphorus in the presence of soluble Pb and U (Fomina *et al*., 2008; Liang *et al*., 2015, 2016a,b,c). Metal phosphates may also result from the presence of inorganic phosphorus sources such as when fungi solubilize phosphate‐containing minerals. A *Penidiella* sp. from an acidic abandoned mine location was capable of accumulating rare earth elements such as dysprosium (Dy; Horiike and Yamashita, 2015). Microbially mediated carbonate precipitation has been used for metal and radionuclide bioremediation, soil stabilization and the reinforcement of concrete structures, but also provides a promising method for the biorecovery of toxic or valuable metals, e.g. Co, Ni and La (Li *et al*., 2015; Kumari *et al*., 2016). Metal carbonates have several industrial applications and are also precursors for important metal oxides, some of which possess electrochemical properties (Li, Q. *et al*. 2016).

Some biomineralization phenomena result in the formation of nanoparticulate forms. The use of metal‐transforming microbes, including fungi, for production of nanoparticles may allow some control over size, morphology and composition. This is relevant to the production of new advanced biomaterials with applications in metal and radionuclide bioremediation, metal biorecovery, antimicrobial treatments (e.g. nanosilver), solar energy, electrical batteries and microelectronics (Lloyd *et al*., 2008; Hennebel *et al*., 2009; Rajakumar *et al*., 2012; Li, Q. *et al*. 2016). Many fungi precipitate nano‐elemental forms of metals and metalloids through bioreduction, e.g. Ag(I) reduction to elemental silver Ag(0); selenate [Se(VI)] and selenite [Se(IV)] to elemental selenium [Se(0)]; tellurite [Te(IV)] to elemental tellurium [Te(0)]. Many of the fungal biominerals mentioned previously can be nanoscale or microscale, which imparts additional properties apart from metal sequestration. For example, mycogenic Mn oxides can sequester metals like Pb, Zn, Co, Ni, As and Cr and also oxidize certain organic pollutants. Many fungi produce metal oxalates on interacting with a variety of different metals and metal‐bearing minerals, e.g. Ca, Cd, Co, Cu, Mg, Mn, Sr, Zn, Ni and Pb (Fomina *et al*., 2005b; Gadd *et al*., 2014) and these have various industrial uses as well as providing another metal biorecovery mechanism.

## Fungi‐metalloid biorecovery

Metalloids can be transformed by fungi through oxidation, reduction, methylation and dealkylation (Gadd, 1993). Two major transformation processes for metalloid biorecovery are the reduction in metalloid oxyanions to elemental metalloids, and methylation of metalloids, metalloid oxyanions or organometalloids to volatile methyl derivatives. It seems that bioreduction is of most potential for biorecovery as only small amounts may be released by volatilization, although this process has been successfully employed for *in situ* bioremediation of contaminated soils and sediments over the longer term. Research has mainly focused on antimony, tellurium and selenium (Jenkins *et al*., 1998; Chasteen and Bentley, 2003; Wilson *et al*., 2010; Pierart *et al*., 2015; Terry *et al*., 2015; Li, Q. *et al*. 2016). Research with arsenic primarily is in the context of detoxification and bioremediation (Mukhopadhyay *et al*., 2002; Loukidou *et al*., 2003) with less on biomining (Sklodowska, 2013). In contrast to bacterial systems, fungal reduction in metalloids has received less attention although many species have the ability to reduce selenium and tellurium oxyanions into elemental forms. For example, *Alternaria alternata* (Sarkar *et al*., 2012), *Phanerochaete chrysosporium* (Espinosa‐Ortiz *et al*., 2015) and *Lentinula edodes* (Vetchinkina *et al*., 2013) were able to generate selenium nanoparticles from reduction of either selenate or selenite, while a *Fusarium* sp., *Penicillium citrinum* (Gharieb *et al*., 1999), *Saccharomyces cerevisiae* (Ottosson *et al*., 2010) and *Rhodotorula mucilaginosa* (Ollivier *et al*., 2011) can produce nanoscale elemental tellurium from tellurite. *Phanerochaete chrysosporium* can also produce mixed Se‐Te nanoparticles when grown with selenite/tellurite (Espinosa‐Ortiz *et al*., 2015, 2017).

## Challenges, limitations and conclusions

Through their properties of metal and mineral bioprecipitation and/or transformation, microorganisms have already demonstrated significant potential for metal(loid) biorecovery. Fungi share many mechanisms with bacteria with some useful differences related to their aerobic chemoheterotrophic metabolism and filamentous branching lifestyle. An ongoing challenge for industry is the development and application of efficient, low‐cost and environmentally friendly methods, and bioprocessing is seen as an important part of the suite of approaches used for metal(loid) recovery. However, limitations lie in large‐scale industrial application and commercial considerations, as well as acceptance by industries strongly rooted in conventional metallurgy, mining and chemical engineering disciplines. Further research is required to optimize the chemical, physical and biological conditions for sustained and effective metal biorecovery from often complex solutions or leachates at extremes of pH and containing other metals, competing anions and chelating agents. Further efforts are also required to integrate biotechnological approaches with conventional hydrometallurgy, mineralogy and abiotic leaching and physicochemical treatments (Vaughan *et al*., 2002). Integration of fungal and bacterial systems is also an area ripe for exploitation as differing mechanisms of bioleaching between bacteria and fungi may have different efficiencies depending on the substrate. Fungal‐mediated bioprecipitation mechanisms may also be applied to bacterial leachates, for example. Metal sulfide bioprecipitation is a successful process mediated by anaerobic sulfate‐reducing bacteria of considerable biotechnological success in a bioremediation context (Hockin and Gadd, 2007). Aerobic fungal processes include formation of oxides, carbonates, phosphates and oxalates, as well as elemental forms of certain metals and metalloids. It is conceivable that anaerobic and aerobic processes might be integrated in certain contexts, as they have in bioremediation. It should also be stressed that microbial metal biorecovery methods can result in production of novel biominerals, which may be of nanoscale dimensions. This provides added value because of the additional physicochemical properties that nanoparticles possess (Lloyd *et al*., 2008; Hennebel *et al*., 2009; Rajakumar *et al*., 2012). With current concern over the security and supply of world metal and mineral resources, it can be concluded that fungal capabilities may offer a potentially useful contribution to biotechnological and physicochemical methods for metal recovery.

## Conflict of interest

None declared.
